# An early and mysterious histerid inquiline from Cretaceous Burmese amber (Coleoptera, Histeridae)

**DOI:** 10.3897/zookeys.733.23126

**Published:** 2018-02-01

**Authors:** Michael S. Caterino, David R. Maddison

**Affiliations:** 1 Department of Plant & Environmental Sciences, Clemson University, Clemson, SC 29634; 2 Department of Integrative Biology, Oregon State University, Corvallis, OR 97331

**Keywords:** amber fossil, Upper Cretaceous, phoresy, inquiline

## Abstract

We describe a new genus and species of Histeridae from Upper Cretaceous Burmese amber, *Amplectister tenax* Caterino & Maddison, **gen. & sp. n.** This species represents the third known Cretaceous histerid, which, like the others, is highly distinct and cannot easily be placed to subfamily. It exhibits prosternal characters in common with Saprininae, but other characters appear inconsistent with this possibility. The abdominal venter is strongly concave, and the hind legs are enlarged and modified for grasping. We hypothesize that this represents the earliest example in Histeridae of modifications for phoresy on social insects.

## Introduction

The early diversification of the beetle family Histeridae is poorly understood. Phylogenetic relationships among extant taxa have been difficult to resolve (Caterino and Vogler 2002, McKenna et al. 2015), and the family’s fossil record is sparse and poorly documented (Chatzimanolis et al. 2006, Caterino et al. 2015). This uncertainty has hindered studies of ecomorphological evolution, which has followed several distinct and repeated trajectories in the family (Caterino and Vogler 2002). The evolutionary pathways taken by histerid lineages have yielded obligate symbioses with diverse animals, including mammals, birds, and, most spectacularly, with social insects. Many of these obligate inquilines show distinctive suites of morphological characters that facilitate their symbioses (Hölldobler and Wilson 1990), including trichomes, exaggerated development of certain body parts, and defensive modifications. Histeridae as a whole is characterized by a body form and structures that give them an ability to defend themselves against attack, including retraction and protection of appendages. This hints at some early symbioses, although there is little support for this in the existing fossil record.

Recent work has begun to reveal a much greater diversity of early Histeridae than previously suspected. Until quite recently the family’s fossil record extended no more than about 40 MYBP (Szwedo and Sontag 2009), but discoveries in Cretaceous Burmese amber have more than doubled this minimum age for the family. Poinar and Brown (2009) described the first of these, *Pantostictus burmanicus*, although the specimens were rather poor and the placement of this species remains unclear. Caterino et al. (2015) described the much better preserved *Cretonthophilus tuberculatus* from the same deposits, hypothesizing placement in Onthophilinae. Here we describe a new genus and species of fossil histerid from the same Burmese amber deposits (with a presumed age of about 99 MYBP, Shi et al. 2012), which offers further insight into the family’s earliest history. This species exhibits distinct hallmarks of inquilinism, with an abdominal-metathoracic leg complex clearly adapted for grasping.

## Methods

**Figure 1.**
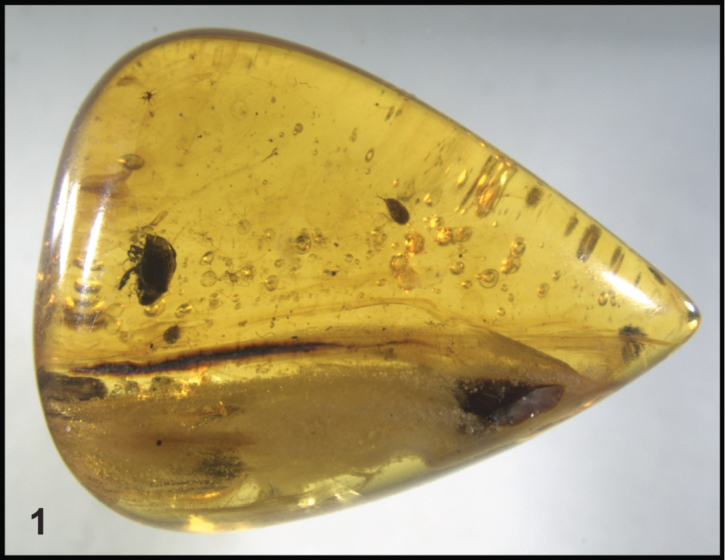
Photograph of original piece of amber (OSAC_0002900057) containing holotype before cutting and polishing.

The original piece of amber (Fig. 1; OSAC lot number OSAC_AMB0000057) was cut into three pieces, and polished. In one piece is the histerid described here (specimen OSAC_0002900057); the remaining pieces contain the other synclusions described below. Photographs were taken using Visionary Digital’s Passport II imaging system (based on a Canon 6D SLR with 65 mm MP-E 1-5× macro lens). Image stacking was done using Helicon Focus (www.heliconsoft.com). Drawings were penciled by hand, traced on a drawing pad, and ‘inked’ in Adobe Illustrator. Measurements were taken using a Leica M125 calibrated eyepiece micrometer.

## Systematic paleontology

### Family: Histeridae Gyllenhal, 1808

#### Subfamily: *incertae sedis*

##### Amplectister

Taxon classificationAnimaliaColeopteraHisteridae

Caterino & Maddison gen. n.

http://zoobank.org/4D931E23-8F6B-4AC6-94C7-3E5229DE3BD2

###### Type species.

*Amplectister tenax* Caterino & Maddison, sp. n.

###### Diagnosis.

**Figures 2–5.**
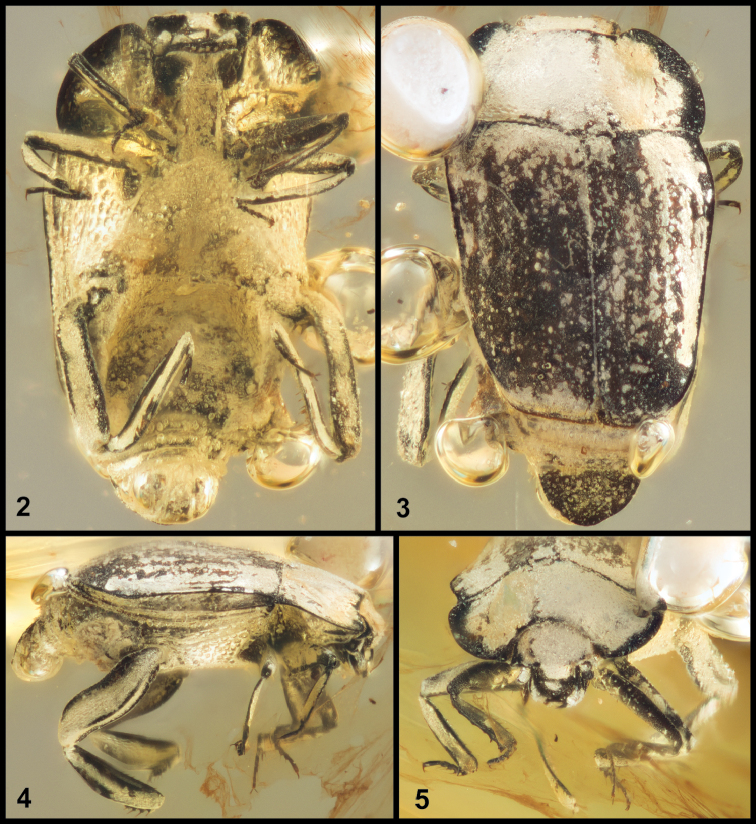
Photographs of holotype. **2** Ventral view **3** Dorsal view **4** Lateral view **5** Frontal view.

**Figures 6–10.**
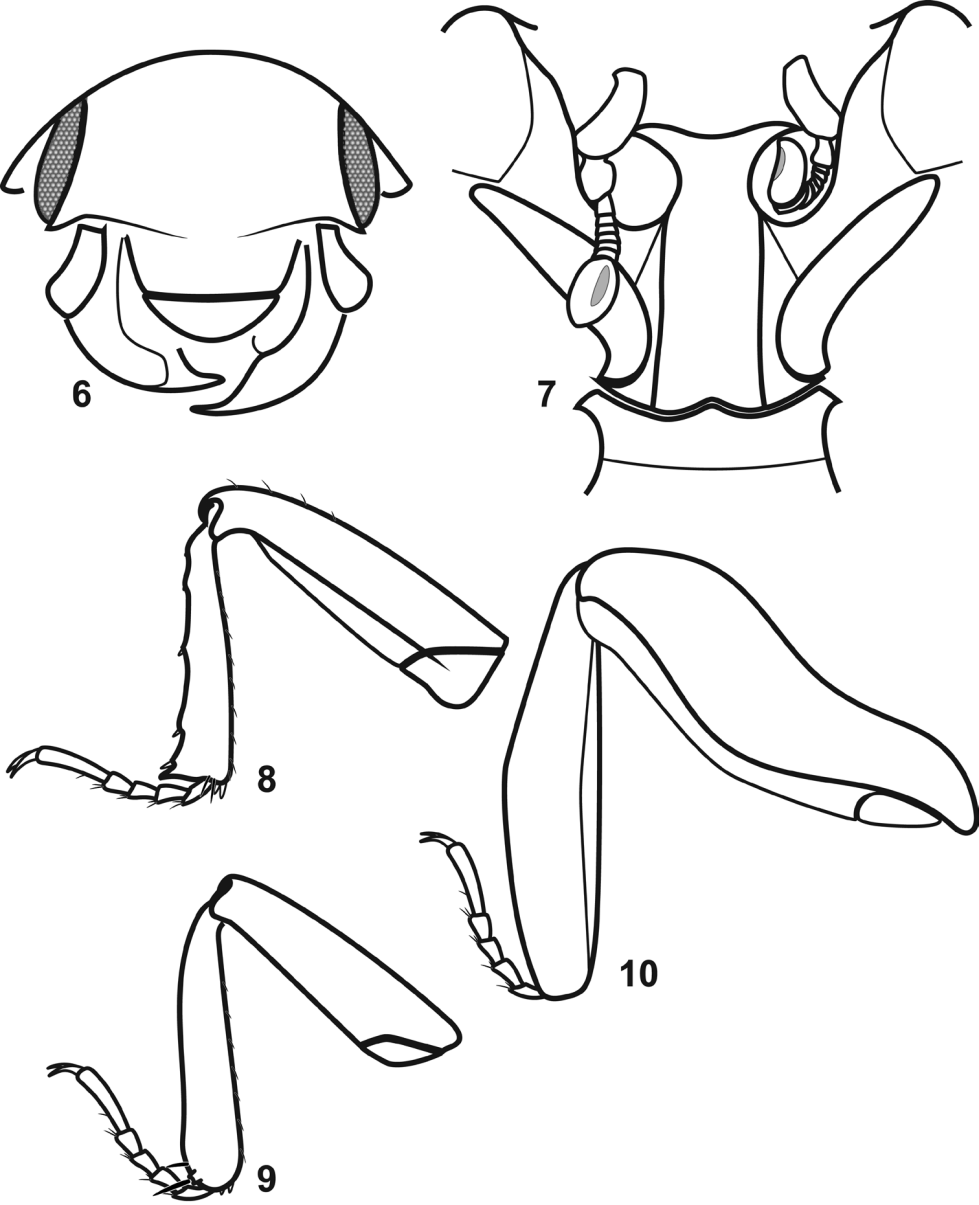
Drawings from holotype. **6** Frontal view **7** Prosternum and antennae **8** Prothoracic leg, anterior view **9** Mesothoracic leg, anterior view **10** Metathoracic leg, anterior view.

Many features distinguish this extinct genus: overall body form quite elongate and flattened (Figs 2–4); frons laterally carinate and projecting over the antennal insertions (Figs 5–6); pronotum with sinuate posterior margin and broadly arcuate lateral margin that is not aligned with the elytral margin (Fig. 3); elytron with two submarginal epipleural carinae (diverging from the posterior pronotal corner; Fig. 4); abdomen deeply concave (Fig. 2); posterior femora and tibiae enlarged and adapted for grasping (Figs 4, 10).

###### Derivation of name.

The genus name (masculine) means ‘the hugging Hister’, referring to its modifications for grasping, from the Latin *amplexus*.

##### Amplectister tenax

Taxon classificationAnimaliaColeopteraHisteridae

Caterino & Maddison sp. n.

http://zoobank.org/A06D06E6-52F1-44F0-84A9-CC648422D095

###### Type material.

Holotype specimen, of unknown sex; type locality: Northern Myanmar: probably Hukawng Valley, collected in 2016; deposited in Oregon State Arthropod Collection, specimen OSAC_0002900057. The specimen was purchased by DRM from Yanling Ying in January 2017. Most of his specimens are from the Noije Bum mine or nearby, Kachin State; a few are from around Nam Sakhaw in Sagaing Division (NW of Haungpa); fewer are from elsewhere in other areas in Kachin State.

###### Description.

Many body surfaces encrusted with thin off-white granular substance and/or thin film of air; textures and surface sculpture difficult to assess. An oblique planar fracture below the anterior part of the body distorts some observations of ventral anterior structures.

Total body (pronotum + elytra) length: 1.41 mm; maximum (humeral) width: 1.02 mm (for all measurements see Table 1). Body surfaces all apparently finely granulate, matte, possibly finely reticulate, not shiny; dorsal surface lacking obvious punctures; ventral surfaces distinctly punctate on most surfaces.

Frons broad, anteriorly prominent (Figs 5–6); eyes present, large, located on sides of head; longitudinal supraocular ridges projecting anterad eyes, continued mediad by prominent, slightly oblique frontal ridges over antennal and mandibular insertions, frontal ridges possibly continuous medially (obscured); frontoclypeal suture not apparent (probably absent, but obscured); epistoma convex along longitudinal midline; labrum evenly rounded apically, convex, without major setae (though with short setal fringe around edges appressed to mandibles); mandibles apically acute, incisor edges short, neither with secondary tooth, left mandible overlapping right in repose; outer surface of mandibles weakly concave in basal half; head mostly retracted, ventral mouthparts not visible. Antennal scape short, expanded slightly to apex, bearing two elongate setae near apex; pedicel about one-third length of scape, subcylindrical; antennal funicle apparently with 6 more or less transverse antennomeres, gradually widening distad, with antennomere 8 nearly as wide as club; antennal club slightly elongate oval, weakly truncate apically, setose, bearing specialized setose patch on inner apical surface (Fig. 7), outer surface may be lightly sclerotized; antennal annuli not apparent.

Pronotum (Figs 3, 5) rather broad, with deep anterior emargination; sides broadly rounded, distinctly widened from obtuse basal corners, widest about one-fourth from base, converging arcuately to rounded anterior corners; central part of pronotal disk convex, lateral margins depressed to broadly explanate, particularly in anterior corners, edges flattened, slightly reflexed.

Scutellum present, small, triangular; elytra (Fig. 3) broad, apparently asymmetrical (possibly optical distortion), the right tapered to a narrower apex than left, moderately flattened, lacking distinct striae but with weak serial depressions, posterolateral corners broadly rounded, apices truncate; each elytron with prominent marginal carina delimiting epipleuron extended from humeral corner around posterior corner, though not attaining apical midline; epipleuron (Fig. 4) with secondary carina extending from humeral corner about two-thirds epipleural length, there merging with lower elytral margin; elytral margin not carinate; metathoracic wings present (protruding slightly beneath posterolateral corner of left elytron).

Propygidium (Fig. 3) exposed, wide, short, bearing numerous stiff setae (this is the only exposed sclerite for which this is true); pygidium subtriangular, with rounded sides and apex, disk depressed with a continuously elevated marginal carina; pygidium slightly opened, but genitalia obscured by air bubbles, sex undeterminable.

Prosternum (Figs 2, 7) elevated at middle, anteriorly incised on either side of keel for passage of antennal funicle, with deep rounded depressions along keel and behind prosternal lobe for reception of antennal club; prosternal keel shallowly emarginate at base, keel elevated, with two prominent carinae, parallel from base to near apex, converging slightly above antennal cavities, distinctly depressed between; very short lateral carinae descend from inner anterior edge of profemur to join keel carinae behind antennal cavity; prosternal lobe minimal, forming broad flange delimiting front of antennal cavities, weakly emarginate where mandibles rest. Hypomeron broadly expanded laterally, with oblique longitudinal carina from anterior corner to near outer corner of profemoral insertion.

Mesoventrite (Figs 2, 7) broad, anterior margin sinuate, weakly but distinctly produced at middle; mesometaventral suture apparently impressed (obscured); metaventrite with prominent, oblique postmesocoxal carinae extending from inner corners of mesocoxae to middle of metacoxa; middle of mesoventrite increasingly depressed posterad; laterally, mesepimeron, metepisternum, and metepimeron all distinct, apparently covered with large punctures (somewhat obscured), as is lateral portion of metaventrite.

Abdominal venter (Fig. 2) deeply concave medially; sides of first ventrite elevated behind metacoxae, forming a distally setose lateral flange; subsequent ventrites transversely depressed, with abdomen deeply arched to pygidial apex.

Legs (Figs 8–10): Procoxa moderately and obliquely transverse; protrochanter subquadrate, with inner corner prominent, setose; profemur narrowed to apex, with anterior, upper edge straight, inner edge weakly excavate for reception of protibia, inner posterior edge weakly expanded bearing few prominent setae; protibia narrow at base, widened weakly to apex, with two small apical spurs at inner corner, laterally with weakly bispinose apex, three to four weak denticles bearing small spines basad along margin, inner edge with series of ~8 fine spines; tarsal groove of anterior face of protibia poorly if at all developed; protarsomeres 1–4 short, subequal, bearing pair of ventral spines, apical tarsomere about three times length of tarsomere 4, with two ventral spines along midline, with pair of regular tarsal claws. Mesocoxa rounded; mesofemur narrowed to apex, with few prominent setae along anterior inner edge; mesotibia narrow, with weak apical spurs, outer edge slightly rounded, with single prominent spine at outer apical margin; outer posterior edge weakly grooved to receive tarsus; tarsus as for protarsus. Metacoxae rounded, widely separated; metatrochanter small, obscured, inserted at posterolateral corner of coxa; metafemur broad and thick, with prominent carinae along inner medial, outer medial, and dorsal margins (narrowly triangular in cross-section), inner surface weakly concave for reception of inner edge of metatibia; metatibia broad and flat, inner margin straight and bearing series of fine spines, outer margin rounded, smooth, inner surface with diffuse cluster of stiff setae about one-third from tibial base; metatarsus segmented as for meso- and protarsus, apparently received along apical half of outer edge of medial tibial face.

**Table 1.** Body measurements in millimeters.

Measurement	mm
Pronotum+elytral (PE) length	1.41
Pronotal length	0.41
Pronotal width	0.98
Elytral length	1.00
Humeral width	1.02
Propygidium length	0.10
Pygidium length	0.24
Head width	0.37
Prosternum length	0.33
Mesoventrite length	0.10
Metaventrite length	0.37
Profemur length	0.35
Protibia length	0.29
Mesofemur length	0.47
Mesotibia length	0.43
Metafemur length	0.73
Metatibia length	0.57

###### Derivation of specific epithet.

The species name means tenacious, referring to its grasp, from the Latin *tenax*.

#### Synclusions

In the same piece of amber as the original specimen were one beetle of the family Eucinetidae (Fig. 1), one mite, and a “stellate hair”, presumably of plant origin. The mite specimen was destroyed in cutting and polishing.

## Discussion

Histerid systematics has relied heavily on the form of the prosternum for classification and phylogenetics (Wenzel 1944; Kovarik and Caterino 2016). This new taxon appears very similar to Saprininae in prosternal characters. Modern Saprininae have a nearly identical form of antennal retraction, with an anterior prosternal notch through which the funicle passes, and a deep anterior depression along the side of the prosternal keel for reception of the club. Looking only at these characters this genus could easily be placed in Saprininae, and even close to a genus such as *Gnathoncus* Jacquelin-Duval. Furthermore, the apparently setose sensory area on the inner surface of the antennal club is strongly suggestive of what in modern Saprininae is termed ‘Reichardt’s organ’, a complex of antennal sensory openings and surfaces (Lackner 2010). Indeed, these prosternal and antennal characters together represent the main morphological synapomorphies of Saprininae (Lackner 2014). However, homology of these characters is not certain, and in numerous other characters *Amplectister* differs substantially from any modern Saprininae. The projecting frontal margin is not known among modern Saprininae. A much weaker form is seen in *Cretonthophilus*, suggesting this could be a plesiomorphy. The elytral striae in *Amplectister* are vaguely impressed, but do not show the highly characteristic saprinine set of elytral striae, with the fourth stria arched to the sutural stria. This isn’t recognized as a Saprininae synapomorphy by Lackner (2014), but may be. *Amplectister* exhibits an emarginate prosternal keel, while that in most modern Saprininae extends as a thin laminate projection over the anterior point of the mesoventrite. This has not been formally evaluated in saprinine phylogeny. Finally, *Amplectister* lacks labral setae, which are universal (though symplesiomorphic) in extant Saprininae. All things considered, it is conceivable that *Amplectister* represents a stem lineage, possessing some but not all apomorphies of extant Saprininae. This possibility merits further exploration and a more quantitative analysis. Deeper examination of *Amplectister* internal characters through micro-CT scanning (e.g. Perreau and Tafforeau 2011, Riedel et al. 2012) would be particularly informative, as some Saprininae apomorphies are found in the genitalia (Lackner 2014).

*Amplectister* shows some similarities to the recently described *Cretonthophilus*, sharing short subpyramidal antennal scapes, frontal carinae, concave sides of mandibles, subdepressed body form, elytral and pronotal lateral margins not colinear, and epipleurae carinate, as well as various features of the legs (profemora able to receive protibia, all tibiae flattened, weakly expanded apically, with spines along inner margins). However, our limited understanding of early histerid phylogeny cannot yet distinguish whether any of these could be synapomorphies of the two. Furthermore, significant differences are numerous. The form and manner of reception of the antennal club on the prosternum is very different, with *Cretonthophilus* having a hypomeral cavity far removed from the prosternal lobe. The form of the antennal club itself is also quite different, with that of *Cretonthophilus* showing deep and distinct sutures between the club’s three antennomeres. *Cretonthophilus* also has an elongated prosternal lobe, and distinct protibial grooves for reception of its protarsus. These phylogenetically compelling characters suggest that *Cretonthophilus* and *Amplectister* occupy distinct branches of early histerid phylogeny. Regarding possible similarities with *Pantostictus burmanicus*, very little can be said due to the lack of phylogenetically informative characters originally described, or visible in the type specimens, which we have recently examined.

The remarkable ventral modifications of *Amplectister* seem clearly adapted for grasping. Grasping in insects serves several purposes and takes a variety of forms. It seems unlikely that the purpose in *Amplectister* is for grasping prey, since in other insects that grasp prey the raptorial modifications are on anterior portions of the body (e.g., in mantises, mantispids, and various Heteroptera), whereas in *Amplectister* the grasping structures are on posterior regions of the body. Some insects show modifications for grasping various substrates, to resist removal by predators, or to prevent being dislodged (elongate legs and enlarged tarsal claws in lotic systems, for example). As the grasping modifications involve only the hind legs in *Amplectister*, rather than all legs, this also seems unlikely.

The posterior location of these modifications on the body suggest courtship as another possible function, and in many insects males exhibit grasping modifications for retaining hold and position on a mate (e.g. Arnqvist 1989, Miller 2003). In some histerids this often includes some degree of concavity on the venter (Caterino and Tishechkin 2013), though invariably on the metaventrite, and none to the extreme seen in *Amplectister*. However, if such a modification were to facilitate mate-holding, we would expect it to correspond more closely in shape to some part of a similar-shaped female. It is not obvious that it does. Also, it is not clear what purpose the distinctive setose brushes on either side of the abdominal concavity would have in mate-holding, nor what role the large and complicated metathoracic legs would play. Mate-holding as the function of these modifications thus seems unlikely.

We suggest instead that the most likely explanation is related to some form of inquilinism. Histeridae exhibit a variety of symbiotic relationships with other organisms, as obligate inhabitants of bird and mammal nests, as well as guests in ant and termite colonies (Kovarik and Caterino 2016). Many insect inquilines grasp their hosts. In the case of vertebrate hosts, many phoretic and parasitic inquilines show modifications for holding on to the fur, feathers, or other more specific parts of their hosts’ bodies. In beetles, the modifications in most such species involve the tarsi (Philips 2011). No such relationships have been described for histerids, but some extant species exhibit chelate tarsi (though not yet directly connected with vertebrate phoresy). Some ant inquilines among Histeridae are known to cling to their host, including the army ant (*Eciton* spp.) guest haeteriines *Nymphister* Reichensperger, which grasps a worker ant’s petiole with its mandibles (von Beeren and Tishechkin 2017), and *Pulvinister* Reichensperger, which rides on the underside of major workers’ heads (Rettenmeyer 1961). This has also been observed in the chlamydopsine *Chlamydopsis loculosa* Lea, which grasps the thorax of its host (*Rhytidoponera* spp.) with its legs (McMillan 1950). Given the presence of setal projections (possible trichomes) on the abdominal concavity of *Amplectister*, and the unusual leg modifications, a social-insect grasping mechanism seems like a reasonable hypothesis. Although it seems unlikely that any specimens will come to light that will allow us to directly test this, improved resolution of basal histerid phylogeny will permit more detailed phylogenetic assessments of the morphological evolution of all these structures and potentially their relationship to function in early symbioses.

## Supplementary Material

XML Treatment for Amplectister

XML Treatment for Amplectister tenax
